# Case report: Post-COVID new-onset neurocognitive decline with bilateral mesial-temporal hypometabolism in two previously healthy sisters

**DOI:** 10.3389/fped.2023.1165072

**Published:** 2023-05-26

**Authors:** Fabrizio Cocciolillo, Daniela Pia Rosaria Chieffo, Alessandro Giordano, Valentina Arcangeli, Ilaria Lazzareschi, Rosa Morello, Giuseppe Zampino, Piero Valentini, Danilo Buonsenso

**Affiliations:** ^1^ Dipartimento di Diagnostica per Immagini, Radioterapia Oncologica ed Ematologia, UOC di Medicina Nucleare, Fondazione Policlinico Universitario Agostino Gemelli IRCCS, Rome, Italy; ^2^ Clinical Psychology Unit, Fondazione Policlinico Universitario A Gemelli IRCCS, Rome, Italy; ^3^ Department of Woman, Children and Public Health, Catholic University of Sacred Heart, Rome, Italy; ^4^ Sezione di Medicina Nucleare, Dipartimento di Scienze Radiologiche ed Ematologiche, Università Cattolica del Sacro Cuore, Rome, Italy; ^5^ Department of Woman and Child Health and Public Health, Fondazione Policlinico Universitario A. Gemelli IRCCS, Rome, Italy; ^6^ Centro di Salute Globale, Università Cattolica del Sacro Cuore, Roma, Italy

**Keywords:** COVID-19, long COVID, children, post-COVID condition, SARS-CoV-2

## Abstract

**Background:**

Long coronavirus disease (COVID) is increasingly recognized in adults and children; however, it is still poorly characterized from a clinical and diagnostic perspective, particularly in the younger populations.

**Case presentation:**

We described the story of two sisters—with high social and academic performance before their severe acute respiratory syndrome coronavirus 2 (SARS-CoV-2) infection—who reported severe neurocognitive problems, initially classified as psychologic pandemic distress and eventually found to have significant brain hypometabolism.

**Conclusions:**

We provided a detailed clinical presentation of neurocognitive symptoms in two sisters with long COVID associated with brain hypometabolism documented in both sisters. We believe that the evidence of objective findings in these children further supports the hypothesis that organic events cause persisting symptoms in a cohort of children after SARS-CoV-2 infection. Such findings highlight the importance of discovering diagnostics and therapeutics.

## Background

The outcomes of severe acute respiratory syndrome coronavirus 2 (SARS-CoV-2) infection are not restricted to acute coronavirus disease 2019 (COVID-19) (e.g., survival vs. death), but there is increasing understanding that patients can develop a wide range of postacute and long-term consequences (1). Long COVID (or post-COVID condition or post-acute COVID syndrome) is among the most discussed outcomes. The post-COVID-19 condition occurs in individuals with a history of probable or confirmed SARS-CoV-2 infection, usually 3 months from the onset of COVID-19 with symptoms lasting at least 2 months that cannot be explained by an alternative diagnosis. Common symptoms include fatigue, shortness of breath, cognitive dysfunction, and others, which generally impact everyday functioning. Symptoms may be new onset following initial recovery from an acute COVID-19 episode or persist from the initial illness. Symptoms may also fluctuate or relapse over time. These symptoms have a negative impact on daily routine (2). Long COVID has also been widely and globally reported in children, although several unknowns remain.

In terms of pediatric long COVID, several clusters of signs and symptoms have been reported, including neurocognitive symptoms (3–7). There has been a significant debate about whether these symptoms are a consequence of psychological distress due to social restrictions implemented during the pandemic or organic consequences of the infection. Several studies have documented objective brain changes in adults with post-COVID neurocognitive symptoms (8–10), while only two reports using brain positron emission tomography (PET) in children have been described (11, 12). However, in these reports, there is a lack of detailed clinical and neurocognitive assessment of children, which limits the ability of clinicians and policymakers to be fully aware of the spectrum of post-COVID neurocognitive symptoms.

In this report, we describe the story of two sisters—with high social and academic performance before their SARS-CoV-2 infection—who reported severe neurocognitive problems, initially classified as psychologic pandemic distress, and eventually found to have significant brain hypometabolism.

## Case presentation

Patient 1 and Patient 2 are two sisters, 13 and 11 years old, respectively, previously vaccinated with two doses of mRNA SARS-CoV-2 vaccine. They got a mild SARS-CoV-2 infection in March 2022 when no restrictions were applied in Italy, masking was not mandatory anymore, and Omicron was the prevalent wave. The infection was characterized by a low-grade fever for 1 day and a headache. The sisters were symptom-free for 3 weeks; afterward, both developed extreme fatigue, joint pain, and cognitive/memory problems. Both were skilled at playing piano and had high rates at school. However, they complained that they could no longer play songs they played routinely. This situation was also confirmed by their piano teacher. Similarly, at schools, they started presenting with memory blackouts and, during maths, could not do easy additions and multiplications. Teachers became concerned and talked to the parents, and both sisters were referred to an outpatient psychologist for psychological support since the presence of symptoms led them to consider ongoing psychological issues, including emulation of the younger one. Six months later, given the lack of improvement, their family pediatrician referred both sisters to our post-COVID outpatient unit.

As part of our protocol (13, 14), children receive a personalized approach targeted to main active problems. For children having persistent unexplained symptoms 90 days after SARS-CoV-2 infection, we perform routine tests to exclude possible alternative diagnoses. If they are excluded, we further evaluate the case according to the main presenting symptoms, which in this case were neurocognitive. Therefore, both sisters underwent routine blood tests, screening for autoimmune diseases, electroencephalography, brain MRI, neurological assessment, and ocular examination, which were all normal. For these reasons and given the persistence of the symptoms, in agreement with the parents, we performed brain PET and neuropsychological assessment with clinical behavioral observation.

The two patients are part of a prospective, personalized follow-up study approved by the local ethics committee (Ethical approval ID4518, Prot0040139/21). Informed consent was obtained from the parents/legal guardians of the participants.

## Brain 18F-FDG PET/CT scans

Brain [18F]-fluorodeoxyglucose positron emission tomography/computed tomography (18F-FDG PET/CT) scans were performed using a Vision 600 PET/CT scanner (Siemens Healthineers). Both patients fasted for 6 h, and in agreement with the current neuroimaging guidelines (15), 3.7 MBq/kg of 18F-FDG was administered intravenously while subjects were resting, lying in supine positions with their eyes closed in a quiet and dimly lit room. Acquisition started 45 min after the radiotracer injection and lasted 20 min. PET images were reconstructed using an iterative time-of-flight algorithm with CT-based attenuation correction and scatter and random corrections. All scans were acquired and reconstructed with the same technical parameters. After visual analysis, a voxel-wise analysis was performed using freely available Statistical Parametric Mapping 8 (SPM8) software (Wellcome Department of Cognitive Neurology, University College London, United Kingdom). To place images in the standard Montreal Neurological Institute neuroanatomic space (MNI; http://www.bic.mni.mcgill.ca), an MNI-based template of 18F-FDG was used as a reference. Spatially normalized images were smoothed by convolution with an isotropic Gaussian kernel (full width at half-maximum, FWHM = 12 mm). A two-sample *t*-test was used to compare both patients with 19 healthy controls selected from a previously gathered database. Statistical analysis was carried out through an unpaired two-sample *t*-test with contrast set at “−1; 1” to detect regional hypometabolism with respect to the control group. The resulting SPM8[t] maps were obtained using a threshold for statistical significance of *p* < 0.001 (uncorrected), and clusters lower than 120 voxels were not considered.

**Figure 1:**
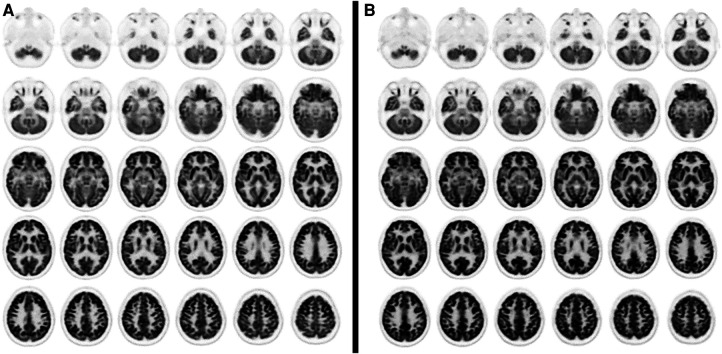
Axial PET slices showing areas of moderate glucose hypometabolism in bilateral mesial brain regions in case 1 (**A**) and case 2 (**B**).

**Figure 2:**
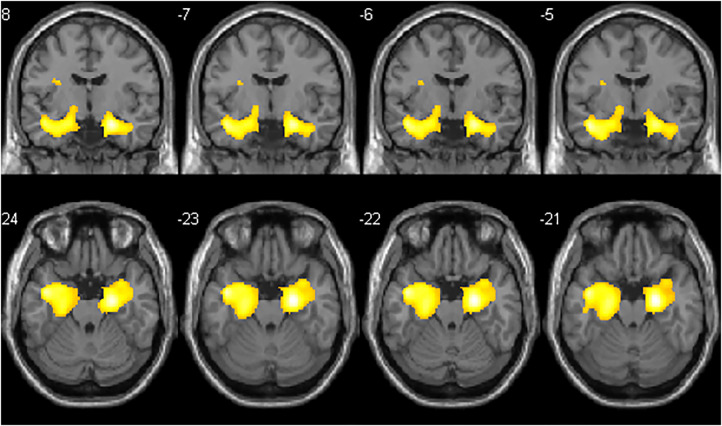
Coronal and axial slices showing SPM areas of reduced brain metabolism superimposed on a T1 axial magnetic resonance reference atlas. The colored areas indicate the locations where the voxel values of both patients are significantly hypometabolic compared to healthy subjects (*P* < 0.001). The *t*-values are represented by the brightness of the color.

Visual analysis of PET images showed a moderate reduction in 18F-FDG uptake in the bilateral mesial-temporal region (Figure 1); these findings were confirmed by the voxel-wise analysis. The SPM8 analysis showed, at a height threshold of *p* < 0.001 (uncorrected), a significant hypometabolism (*p* < 0.001) in both patients (*n* = 2), as compared to healthy subjects (*n* = 19), in the left and right mesial-temporal regions with a peak in the bilateral hippocampus (right: MNI coordinates 22/-12/-20 mm with a T-score of 6.7; left: MNI coordinates −20/−16/−19 mm with a T-score of 6.1; Figure 2).

## Neuropsychological assessment and clinical behavioral observation of P.A.

Methods: A clinical observation was performed. Wechsler Intelligence Scale for Children, Fourth Edition (WISC-IV), Full Scale Intelligence Quotient (FSIQ), Verbal Comprehension Index (VCI), Visual Perceptual Index (VPI), Working Memory Index (WMI), Processing Speed Index (PSI), and academic tests AC-MT, BVSCO-2, and MT for neuropsychological evaluation were administered, i.e., Nepsy II, Bells test, and Beery test for VMI and visual perception.

Furthermore, to obtain a complete psychological profile of the patients, their parents were asked to complete Thomas M. Achenbach's Child Behaviour Checklist Ages 6–18 (CBCL/6–18) questionnaire, from which the following emerged:

### Case 1

Clinical observation: *Clinical behavioral observation:* Patient 1 entered the assessment setting with her primary caregiver. She showed no difficulty during separation and reunion with the same. One can, therefore, assume the existence of a secure attachment style.

Although the girl has been introverted and shy from the familiarization stage, she was consistently cooperative in performing the tests; she also kept continuous and modulated eye contact. Specifically, she remained concentrated on the task at times of greatest cognitive effort.

Her linguistic production was characterized by little ability to initiate conversation spontaneously; in fact, she merely responded to the stimuli proposed by the clinician. However, her language production demonstrated a lexical repertoire appropriate for her chronological age. Good ability in concept formation and retrieval, as well as in problem-solving and verbal reasoning, was also noted. Her morphosyntactic construction and her ability to use appropriate free and bound morphology were appropriate for her chronological age.

Patient 1's receptive functions were consistent and fully appropriate throughout the assessment. She showed a good ability to decode and retain the tasks in memory for the time required to perform the tests, to which she seemed able to respond fully and nontangentially.

Her levels of attention and vigilance were consistent throughout the screening. Patient 1 seemed able to focus her attention briefly on the information provided by the examiner; she had no difficulty remaining concentrated on the tasks for long periods.

### Cognitive level tests (WISC-IV)

*Drawing cubes:* This test measures the ability to analyze and synthesize abstract visual stimuli by grasping their spatial relationships (p.p. 12, within the normal range).

*Similarities:* This test gives an estimate of verbal reasoning and level of concept formation, also involving language development, lexical knowledge, auditory comprehension, memory, and the ability to discriminate between essential and nonessential features (p.p. 14, above normal).

*Digit memory:* This test measures short-term auditory memory, working memory, and attention and concentration skills (p.p. 13, within the normal range).

*Illustrative concepts:* This test measures abstract categorical reasoning, inductive reasoning, and general information (p.p. 14, above normal).

*Cipher:* This test measures processing speed, short-term memory, learning ability, visual perception, visuomotor coordination, visual scanning ability, cognitive flexibility, attention span, and motivation (p.p. 16, above normal).

*Vocabulary:* This test measures lexical knowledge and verbal concept formation (p.p. 11, within the normal range).

*Letter and number reordering:* This test assesses skills in mental manipulation, attention, auditory short-term memory, and visual–spatial representation (p.p. 4, below average).

*Reasoning with matrices:* This test gives an estimate of fluid intelligence, particularly inductive reasoning and general sequential reasoning (p.p. 15, above normal).

*Comprehension:* This test assesses verbal reasoning, general information, conceptualization ability, verbal comprehension, and verbal expression ability. (p.p. 13, normal range).

*Symbol search:* This test measures perceptual and processing speed, visual short-term memory, visuomotor coordination, cognitive flexibility, visual discrimination, and concentration ability. (p.p. 11, normal range).

## Academic tests

### New MT Reading tests

In the reading test of the passage “the bow in antiquity”, Patient 1 scored below normal for speed (3.64 syllables per second) but scored in the normal range for correctness (0 errors). Thus, her performance was compatible with a level range corresponding to “Demand for Attention” and “Criterion Fully Achieved”.

On the comprehension test of the passage “So I found 26 thousand euros”, Patient 1 scored in the normal range (9/12 correct answers). Thus, her performance was within the level band “Criterion fully achieved”.

### BVSCO-2 writing and orthographic proficiency assessment battery-2

On the dictation test of the song “The Lions’ Assault”, Patient 1 made 0 errors and scored in the normal range. Thus, her performance was compatible with the level band corresponding to “Criterion Fully Achieved”.

### AC-MT mathematical logical reasoning test

On the test designed to investigate written calculation skills, Patient 1 scored 4/8 and was in the “Demanding Attention” level range.

## Neuropsychological tests

### Memory tests

#### Verbal memory

In tests that required her to recall a list of 16 semantically unrelated words presented five consecutive times, Patient 1's performance was within the normal range for both short-term recall (M.b.T. p.p. 8) and long-term recall (M.l.t. p.p. 9).

Beery test

• On a test of visual perception, her results were in the normal range (p.p. 96).
• On a test of visuomotor integration (VMI), her results were in the normal range (p.p. 93).

#### Tests of visuospatial analysis and attention

On a test that requires identifying and crossing out a figurative stimulus among distractors (i.e., The Bells Test revised, by *Gauthier*), her results were in the normal range for speed of task execution (*z*-score −0.09) and accuracy in the execution of the test (*z*-score −0.46).

#### Frontal tests

On a task in which she was required to produce a list of words according to a criterion provided by the examiner (i.e., the subtest of the NEPSY II batteries), her results were in the normal range both when the required criteria were phonological (“s/f”, p.p. 11) and categorical (“animals/food/drinks”, p.p. 12).

With regard to the nonword repetition test, Patient 1 scored in the normal range (p.p. 11).

## Personality and behavior questionnaire

### CBCL (Child Behavior Checklist for Ages 6–18) by Thomas M. Achenbach

The following results emerged from the completion of the parent-facing questionnaire:

1. On the Internalization scale, which includes the anxiety/depression, withdrawal/depression, and somatic complaints scales, her results were *borderline* (*T* score: 67).
2. On the Externalizing scale, which includes rule transgression behavior and aggressive behavior, her results were in the normal range (*T* score: 51)
3. On the Total Problems scale, which includes all the items that make up the different scales, her results were also in the normal range (*T* score: 54).

The examination showed above-normal cognitive development for the patient's chronological age (FSIQ 119), which was characterized by a disharmonious profile in the presence of a significant difference (>12 points) between the different indices (i.e., VCI: 116; VPR index: 124; WMI: 91; PSI: 121).

With regard to the screening of academic skills, slight immaturities emerged in the area of reading, in terms of speed, and logical-mathematical procedure, compared to the full achievement of skills in passage writing, test comprehension, reading (i.e., in terms of correctness), and text writing.

Regarding the analysis of the neuropsychological tests, scores within the normal range were found in all skills investigated by the instrument.

Finally, regarding completing the questionnaire addressed to the parent, a score in the lower limits of the norm emerged on the Internalization subscale and full achievement emerged on the Externalization and Total Problems subscales. Specifically, borderline scores emerged on the anxiety/depression subscale.

### Case 2 (Patient 2)

Clinical observation: Patient 2 entered the assessment setting accompanied by her primary caregiver; she showed no apparent difficulty during separation or reunion with the same. Therefore, we can assume the existence of a secure type of attachment. She cooperated consistently and also maintained discontinuous eye contact throughout the test.

Her language production was characterized by low communicative intentionality. However, in language production, she consistently demonstrated a lexical repertoire appropriate for her chronological age.

Patient 2's receptive functions seemed adequate to the setting throughout the testing period. She demonstrated a fair ability to decode the tasks and retain them in memory for the time required to perform the tests; in fact, she seemed able to respond sufficiently, completely, and directly.

Her levels of attention and alertness were fairly consistent throughout the screening. Patient 2 seemed able to briefly focus her attention on the information provided by the examiner; however, she also had a little difficulty remaining concentrated on the tasks for long periods.

## Cognitive level tests (WISC IV)

*Drawing cubes:* Her results were in the normal range (p.p. 10).

*Similarities:* Her results were in the normal range (p.p. 10).

*Digit memory:* Her results were *low average* (p.p. 7).

*Concepts illustrated:* Her results were in the normal range.

*Cipher:* Her results were *below* the normal range (p.p. 3).

*Vocabulary:* Her results were in the normal range (p.p. 10).

*Letter and number rearrangement:* Her results were *below* the normal range (p.p. 5).

*Reasoning with matrices:* Her results were in the normal range (p.p. 11).

*Comprehension:* Her results were in the normal range (p.p. 8).

*Symbol search:* Her results were low average (p.p. 7).

## Academic tests

### New MT Reading tests

On the reading test of the passage “*The Wasps*” she scored in the normal range for speed (3.78 syllables per second) and correctness (2 errors). Thus, her performance was compatible with a level range corresponding to “Sufficient performance”.

On the comprehension test of the story “*The Stolen Violin”*, Patient 2 scored in the normal range (8/12 correct answers). Thus, she performed in the “Sufficient Performance” level band.

### BVSCO-2 writing and orthographic proficiency assessment battery-2

On the dictation test of the passage “Little Antelope”, Patient 2 made 0 errors; thus, she scored in the normal range. In fact, her performance was compatible with the level band corresponding to “Criterion fully achieved”.

### Test of logical-mathematical reasoning AC-MT

On the test designed to investigate written calculation skills, Patient 2's performance corresponded to 7/8, i.e., in the “Sufficient performance” level band.

## Neuropsychological tests

### Memory tests

#### Verbal memory

On tests that required recalling a list of 16 semantically unrelated words presented 5 consecutive times, Patient 2 performed *slightly below normal* in short-term recall (M.b.T. p.p. 6) and below *normal* in long-term recall (M.l.t. p.p. 2).

#### Frontal tests

In a task that required producing a list of words according to a criterion established by the examiner (i.e., a subtest of the NEPSY II battery), her results were *borderline* when the required criterion was phonological (“s/f”, p.p. 7) and categorical (“animals/food-drinks”, p.p. 7).

On the repetition of the nonword test, Patient 2 scored *below the normal range* (p.p. 4).

#### Beery's test

• On a visual perception test, her results were in the normal range (p.p. 110)
• On a test of visuomotor integration (VMI), her performance was *borderline* (p.p. 76)

#### Tests of visuospatial analysis and attention

On a test that required identifying and crossing out a figurative stimulus among distractors (i.e., The Bells Test revised, by *Gauthier*), she performed below the norm for speed of task execution (*z*-score −3.28) and accuracy in performing the test (*z*-score −7.15).

## Personality and behavior questionnaire

### CBCL (Child Behaviour Checklist for Ages 6–18) by Thomas M. Achenbach

The following results emerged from the completion of the parent-facing questionnaire:

1. On the Internalization scale, which includes the anxiety/depression, withdrawal/depression, and somatic complaints scales, M.'s results were *below normal* (*T* score: 67).
2. On the Externalization scale, which includes rule transgression behavior and aggressive behavior, the results were in the normal range (*T* score: 51).
3. Patient 2's results were also in the normal range on the Total Problems scale, which includes all the items that make up the different scales (*T* score: 54).

## Three-month follow-up

Currently, both sisters are at 3-month follow-ups. Given the support they received from school and their piano teachers, they have returned to attend school and piano lessons full time. They exhibited a gradual subjective improvement, although they did not yet showed a feeling of having returned to pre-COVID cognitive performance in terms of music skills and ability to retain the information they study at school. A reassessment with a complete neurocognitive evaluation has been participated in summertime since the sisters missed several days of school and parents asked us to posticipate the tests to not miss other days.

## Discussion and conclusions

In this case report, we have described two previously healthy girls, with no risk factors nor family history, with new onset neurocognitive decline after mild SARS-CoV-2 infection. While psychological distress has been initially hypothesized, objective brain hypometabolism and limitation in neurocognitive tasks have been documented, suggesting that some—still uncharacterized—events related to the infection can explain the behavioral changes. The sisters have been enrolled in studies defining proteomic, miRNA, and genetic profiles to better understand the biosignature of long COVID and potential pharmacological targets.

In summary, the examination revealed lower normal cognitive development for chronological age (FSIQ 83), characterized by a disharmonious profile in the presence of a significant difference (>12 points) between the different indices (VCI: 96; VPI: 98; WMI: 76; PSI: 71). With regard to the screening of the academic tests, M. demonstrated full achievement of skills in the passage comprehension test, reading (for both speed and correctness), passage writing, and logical-mathematical procedures. Following the analysis of the neuropsychological tests, below-normal scores emerged for memory, i.e., short-term and delayed memory, and the repetition of nonwords; also, scores in the below-normal limits emerged for word recall in semantic and phonological terms. Finally, in the analysis of The Bells Test revised, below-normal scores emerged for both selective and sustained attention. Regarding completing the questionnaire, M. obtained clinically relevant below-normal scores on the so-called Internalization subscale; by contrast, she scored in the normal range on both the Externalization and Total Problems subscales and obtained clinically relevant below-normal scores on the anxiety/depression subscale.

Neurocognitive deficits are somehow in line with the observed deficits at brain PET. The medial temporal lobe (MTL) includes the hippocampus, amygdala, and parahippocampal regions and is crucial for episodic and spatial memory. MTL memory function consists of distinct processes such as encoding, consolidation, and retrieval (16, 17). Both girls have all these deficits.

These findings are in line with two previous reports showing brain hypometabolism in children with neurocognitive Long COVID and further reinforce the organic nature of the disease (11, 12). The pathogenesis of these events is still unknown, although several new hypotheses are under investigation as new studies provide more insights into possible consequences of SARS-CoV-2 infection. One of the currently most important hypotheses is related to the persistence of SARS-CoV-2 proteins/antigens in the body, including the brain, which may still have uncharacterized biological events. Viral persistence has been widely described in adults, and a recent review has also documented that even in children with mild or asymptomatic infection, SARS-CoV-2 proteins/antigens may persist in several body parts and may potentially trigger local inflammatory processes (18). In line with this hypothesis, a more recent case series documented that two newborns infected during pregnancy had severe parenchymal atrophy and cystic encephalomalacia, and one child had a sudden unexplained episode with evidence of SARS-CoV-2 proteins/antigens in the central nervous system at autopsy (19). Other hypotheses include chronic endotheliopaty with circulating microclots and hypoperfusion (20) and microglial inflammation (21).

Importantly, this report provides more clinical information and detailed neurocognitive findings and examinations compared with the previous reports, supporting clinicians dealing with similar cases in future decision-making and family communication. A complicating factor is that these patients, including our sisters, were perfectly aware of their changes in school and extrascholastic performances, and this limited their interactions, as they were ashamed of attending schools and piano lessons. In this case, recognition of an organic event has been a piece of important information for the parents, teachers, and children as well, confirming that these limitations were real and not pretended actions. Such findings are useful not only to facilitate patients' access to research projects but also to implement supportive tools for the families (e.g., we have been able to provide certificates and informative tools for teachers, friends, and other parents to support the graduate and protected reintroduction of the two sisters at school and piano lessons). Specific treatments are not yet available. So far, several attempts or theories have been made to improve the long COVID neurocognitive symptoms, such as cognitive processing therapy or phenytoin for cognitive deficit and exercise training for functional and psychological problems (22). Other potential therapeutic targets have also been theoretically proposed, including nuclear factor (erythroid-derived 2)-like 2 and oxidative stress, amyloid fibrin microclots, histamine receptors and antagonists, probiotics, microvascular dysfunctions, and cholinergic anti-inflammatory pathways (22). However, none of them provided conclusive results, and none has been studied in children. Therefore, after discussion with the family, we decided to use a product available in Italy that contains magnesium oxide, bulking agent cellulose, turmeric Meriva fitosomas rhizome e.s. titrated at 18% in curcuminoids, zinc gluconate, anticaking agents silicon dioxide, magnesium salts of fatty acids, and carboxymethyl cellulose sodium, soy seeds e.s. 40% isoflavones, 98% resveratrol, selenium, methionine, cholecalciferol, and folic acid. We opted for this treatment based on the published literature showing positive biological effects of selenium, vitamin D, vitamin E, zinc, magnesium, and polyphenols on cognitive functions (23–30). Treatment has been prescribed for 6 months, as, in recent studies, we have found that most symptoms may recover within 6 months in many children infected with the omicron variant (31, 32). Importantly, evidence of brain changes (mostly in function) has been described in the literature (8–12), and our findings add to the available literature the complexity of new-onset neurocognitive problems after SARS-CoV-2 infection.

As similar symptoms affected both sisters, we have performed a multigenic analysis for immunologic and hematologic diseases, which is under investigation.

In conclusion, we described two cases of brain hypometabolism and neurocognitive decline in two sisters, previously healthy before SARS-CoV-2 infection. Given the exclusion of other conditions and the evidence from the literature that long COVID can be associated with such symptoms, we have associated the clinical picture with a scenario of long COVID with a neurocognitive pattern with evidence of brain hypometabolism. We believe that the evidence of objective findings in these children further supports the hypothesis that organic events cause persisting symptoms in a cohort of children after SARS-CoV-2 infection. Importantly, we hope this case series will support other clinicians in the appropriate diagnostic pathway in this circumstance and stimulate the curiosity of researchers and funding agencies to better understand long COVID.

## Data Availability

The original contributions presented in the study are included in the article, further inquiries can be directed to the corresponding author.
